# Accuracy validation of adjuvant! online in Taiwanese breast cancer patients - a 10-year analysis

**DOI:** 10.1186/1472-6947-12-108

**Published:** 2012-09-17

**Authors:** Kuo Yao-Lung, Chen Dar-Ren, Chang Tsai-Wang

**Affiliations:** 1 Department of Surgery, National Cheng Kung University Hospital, College of Medicine, National Cheng Kung University, Tainan and Dou-Liou Branch, 138 Sheng Li Road, Tainan, 704, Taiwan; 2 Comprehensive Breast Cancer Center, Changhua Christian Hospital, 135 Nanxiao St, Changhua, 500, Taiwan; 3 Department of Surgery, National Cheng Kung University Hospital, College of Medicine, National Cheng Kung University, 138 Sheng Li Road, Tainan, 704, Taiwan

**Keywords:** Adjuvant! Online, Breast cancer, Mortality prediction, Taiwan

## Abstract

**Background:**

Adjuvant! Online ( http://www.adjuvantonline.com) is an Internet-based software program that allows clinicians to make predictions about the benefits of adjuvant therapy and 10-year survival probability for early-stage breast cancer patients. This model has been validated in Western countries such as the United States, United Kingdom, Canada, Germany, and Holland. The aim of our study was to investigate the performance and accuracy of Adjuvant! Online in a cohort of Taiwanese breast cancer patients.

**Methods:**

Data on the prognostic factors and clinical outcomes of 559 breast cancer patients diagnosed at the National Cheng Kung University Hospital in Tainan between 1992 and 2001 were enrolled in the study. Comprehensive demographic, clinical outcome data, and adjuvant treatment data were entered into the Adjuvant! Online program. The outcome prediction at 10 years was compared with the observed and predicted outcomes using Adjuvant! Online.

**Results:**

Comparison between low- and high-risk breast cancer patient subgroups showed significant differences in tumor grading, tumor size, and lymph node status (*p* < 0.0001). The mean 10-year predicted death probability in 559 patients was 19.44%, and the observed death probability was 15.56%. Comparison with the Adjuvant! Online-predicted breast cancer-specific survival (BCSS) showed significant differences in the whole cohort (*p* < 0.001). In the low-risk subgroup, the predicted and observed outcomes did not differ significantly (3.69% and 3.85%, respectively). In high-risk patients, Adjuvant! Online overestimated breast cancer-specific survival (*p* = 0.016); the predicted and observed outcomes were 21.99% and 17.46%, respectively.

**Conclusions:**

Adjuvant! Online accurately predicted 10-year outcomes and assisted in decision making about adjuvant treatment in low-risk breast cancer patients in our study, although the results were less accurate in the high-risk subgroup. Development of a prognostic program based on a national database should be considered, especially for high-risk breast cancer patients in Taiwan.

## Background

Breast cancer treatments are based mainly on international guideline recommendations (e.g., the National Comprehensive cancer Network (NCCN) guidelines or St. Gallen consensus) [1-3]. Improvements in the efficacy of adjuvant chemotherapy have also improved the prognosis for early breast cancer [4]. Adjuvant drug therapy can extend survival in breast cancer patients, although the balance between risks and benefits must be considered. The decision to prescribe adjuvant systemic therapy for early breast cancer patients is complex and requires consideration of conditions such as the patient’s and tumor characteristics, treatment effectiveness, and clinician and patient preferences. However, inaccurate outcome predictors can lead to the over- or undertreatment of patients. Recently, several statistical models or programs have been developed to predict outcomes for early breast cancer patients [5-10]. These programs can help to inform patients and allow them to choose a more individualized treatment strategy based on a number of prognostic factors.

Adjuvant! Online is a Web-based, open-access computer program that predicts patient outcome at 10 years in breast cancer patients [10]. The development of the program is based on information from the Surveillance, Epidemiology, and End Results (SEER) registry. The SEER registry includes data for about 10% of breast cancer patients in the USA. The program analyzed the outcome of 10-year survival probabilities, risk of relapse, and survival using patient information and tumor characteristics such as age, tumor size, tumor grade, estrogen receptor (ER) status, and lymph node status [11].

Adjuvant! Online was validated successfully by Olivotto and colleagues in a population-based series of 4083 early-stage breast cancer patients in Canada [12]. They concluded that the Adjuvant! Online program accurately predicted the observed breast cancer-related death outcome within 2% in the whole study population. However, Asian populations might differ from those in the USA or Canada, and the accuracy of Adjuvant! Online in predicting outcomes in the Asian, particularly the Taiwanese population is unknown.

The aims of our study were to validate the accuracy of Adjuvant! Online, to predict outcomes in different patient groups, and to distinguish individuals who will need different prediction methods in a cohort of Taiwanese patients with breast cancer treated at a multidisciplinary tumor board in a university hospital.

## Methods

Between January 1, 1992, and December 31, 2001, 652 breast cancer patients were diagnosed and treated at National Cheng Kung University Hospital (NCKUH) in Tainan, Taiwan. The final cohort involved in the subset of 559 patients fulfilled the following criterion: follow-up period > 10 years or death due to breast cancer within 10 years from the diagnosed date. Ninety-three patients were excluded because of loss to follow-up or death from other causes. In all analyzed cases, 472 patients whose follow-up period was > 10 years were classified as “survived” (84.44%), and the other 87 patients who died of breast cancer within 10 years were classified as “died” (15.56%). Those who died of breast cancer beyond the 10-year follow-up were also classified as survived. Ethical approval was provided by the Human Experiment and Ethics Committee of the National Cheng Kung University Hospital (A-ER-101-013).

The demographic data included age at the time of presentation for cancer, and the pathological findings included tumor size, axillary lymph node status, tumor histological grade, and ER status. All pathological specimens were reviewed by breast pathologists at NCKUH. Tumor size was measured using pathological reports from NCKUH. The Scarff-Bloom-Richardson (SBR) system was used for tumor grading and was based on the following morphological features: nuclear pleomorphism of tumor cells, degree of tumor tubule formation, and tumor mitotic activity. To determine the ER status, immunohistochemistry was performed on formalin-fixed, paraffin-embedded breast cancer tissue samples from the patients. Positive ER status was defined as nuclear staining > 1%. Immunohistochemistry was performed using anti-ER (clone 6 F11, Ventana Medical Systems, Strasbourg, France). Postoperative adjuvant chemotherapy was performed according to the NCCN and St. Gallen guidelines.

Postoperative adjuvant chemotherapy was performed according to NCCN and St. Gallen guidelines. Between the period 1992 to 1995, CMF regimen was used as standard chemotherapy (cyclophosphamide 500 mg/m^2^, methotrexate 50 mg/m^2^ and 5-FU 500 mg/m^2^). Since 1996, FEC regimen (5-FU 500 mg/m^2^, epirubicin 100 mg/m^2^ and cyclophosphamide 500 mg/m^2^) was given in the majority of patients. Since 1998, for high risk patients, TEC regimen (Taxotere 75 mg/m^2^, epirubicin 75 mg/m^2^ and cyclophosphamide 500 mg/m^2^) was performed.

*χ*^2^ and two-sample independent *t* tests were used to compare variables between the two subgroups. Data for demographic and tumor characteristics for each patient, such as age, ER status, grade, tumor size, lymph node status, and treatment modalities (chemotherapy or/and hormone therapy) were entered into the Adjuvant! Online program (version 8.0), which produced a 10-year predicted probability for death due to breast cancer. For the comorbidity item “average for age” was imputed for all patients. The Hosmer–Lemeshow test was used to assess whether the predicted probabilities matched the observed probabilities in subgroups of the patient population [13]. The difference in the number of categories reflects the different subgroup sizes. P values < 0.05 were considered significant. The observed probability was regressed on the predicted probability, and *R*^2^ was calculated to measure the goodness of fit of the linear regression line. All statistical analyses were performed with SPSS 17.0 software.

## Results

The patients’ characteristics and the distribution of the whole patient cohort is described in Table 1. The mean 10-year predicted death probability in 559 patients was 19.44%, and the observed death probability was 15.56% (*p* < 0.001). The predicted event rate evaluated using the Adjuvant! Online formula significantly overestimated the real event rate in the patients in Taiwan. We used the linear regression model to adjust for the difference between the predicted and observed probability by calculating the predicted probability using the Adjuvant! Online formula as the first step, inserting the predicted value into the regression formula, and then estimating the observed probability. Compared with the predicted probability, the estimated observed probability was closer to the real probability. The calculated *R*^2^ was 0.843, showing excellent fit of the regression model.

**Table 1:** Patient characteristics in the whole cohort

	**Total N = 559 (%)**
**Age, years**	**49.5**
**ER**	
Negative	204 (36.5)
Positive	345 (61.7)
Unknown	10 (1.8)
**Grade**	
I	130 (23.3)
II	204 (36.5)
III	105 (18.8)
Unknown	120 (21.5)
**Tumor Size**	
T1	235 (42.0)
T2	285 (51.0)
T3	39 (7.0)
**Node**	
N0	323 (57.8)
N1	135 (24.2)
N2	50 (8.9)
N3	51 (9.1)
**Status**	
Died	87 (15.6)
Survived	472 (84.4)
**Hormone therapy**	
Yes	476 (85.2)
No	83 (14.8)
**Chemotherapy**	
Yes	381 (68.2)
No	149 (26.7)
Less than expected cycles	29 (5.1)

However, in our study, the model was overoptimistic in the high risk patients. We categorized the patients into low- and high-risk subgroups. Patients with all of following prognostic factors were classified as low-risk: aged ≥ 35 years and had a tumor size ≤ 2 cm, negative lymph node status, positive ER/PR receptor status, and tumor grade I. The patients with any one of the following prognostic factors will be classified as high-risk: aged < 35 years or had a tumor size > 2 cm or positive lymph node status or tumor grade II or III or negative ER/PR receptor status. Comparison between the low- and high-risk groups showed significant differences in tumor grade, tumor size, and lymph node distribution (*p* < 0.0001), which justified the division of the patients into the two subgroups. The mean age did not differ significantly between the two groups (*p* = 0.056). (Table 2).

**Table 2:** Patient characteristics in the low- and high-risk subgroups

	**Low Risk (N = 78)**	**High Risk (N = 481)**	***p-*value**
**Age, years**	**51.7 (11.0%)**	**49.1 (11.1)**	**0.056**
**ER**			< 0.0001
Negative	0	192 (39.9)	
Positive	78 (100%)	279 (58.0)	
Unknown	0	10 (2.1)	
**Grade**			< 0.0001
I	78 (100%)	52 (10.8)	
II	0	204 (42.4)	
III	0	105 (21.8)	
Unknown	0	120 (24.9)	
**Tumor Size**			< 0.0001
T1	78 (100%)	183 (38.0)	
T2	0	259 (53.8)	
T3	0	39 (8.1)	
**Node**			< 0.0001
N0	78 (100%)	245 (50.9)	
N1	0	135 (28.1)	
N2	0	50 (10.4)	
N3	0	51 (10.6)	
**Status**			0.002
Died	3	84	
Survived	75	397	
**Hormone therapy**			0.001
Yes	76 (97.4)	400 (83.2)	
No	2 (2.6)	81 (16.8)	
**Chemotherapy**			< 0.0001
Yes	31 (39.7)	350 (72.8)	
No	45 (57.7)	104 (21.6)	
Less than expected cycles	2 (2.6)	27 (5.6)	
Observed probability	3.85%	17.46%	
Predicted probability	3.69%	21.99%	

**Figure 1:**
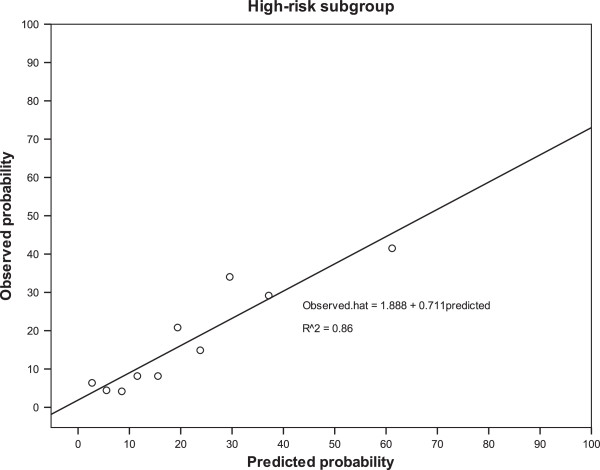
Predicted versus observed outcome in the high-risk breast cancer patients in our patient population.

**Figure 2:**
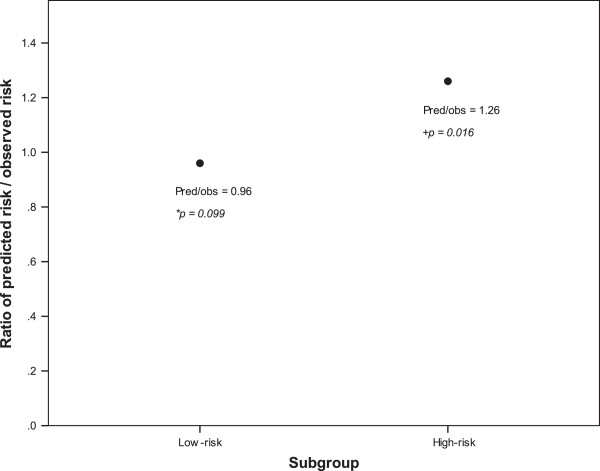
**Ratio of predicted risk to observed risk between the low-risk and high-risk subgroups.** Pred: predicted risk, obs: observed risk. *: the difference between predicted and observed risk was not significant (*p* = 0.099). The Adjuvant! Online model showed a good discriminative performance in the low risk patients. +: the difference between predicted and observed risk was significant (*p* = 0.016). The Adjuvant! Online model showed an overestimation of observed 10-year breast cancer-specific survival in the high risk patients.

In 78 low-risk patients, the mean predicted death probability was 3.69%, which did not differ significantly from the 3.85% observed death probability (ratio of predicted/observed risk = 0.96; *p* = 0.099). The Adjuvant! Online probability accurately predicted the outcomes of patients in the low-risk group. However, the difference between predicted and observed death probabilities was significant in the 481 high-risk patients. A linear regression model was also constructed to adjust for the difference between the predicted and observed probabilities in the high-risk subgroup. The calculated *R*^2^ was 0.86, showing excellent fit of the regression model (Figure 1). The Adjuvant! Online model showed an overestimation of breast cancer-specific survival in Taiwanese high risk breast cancer patients (ratio of predicted/observed risk = 1.26; *p* = 0.016) (Figure 2).

The rate of loss to follow-up was 14.26% because some patients were lost of follow-up after 5 years hormonal treatment. These patients were excluded from our data analysis, and our more conservative data analysis method might have overestimated the death probability.

## Discussion

Adjuvant treatment for postoperative early breast cancer patients remains a great challenge for physicians and patients who must consider both the risks and benefits of treatment, possible comorbidities, and especially the desire to maintain quality of life. Several tools to support decisions have been developed [5,7,8,14,15]. One such tool, Adjuvant! Online, is a computerized, Web-based program that predicts recurrence and mortality risk and the benefit of adjuvant treatment in early breast cancer patients [10]. The program is based on the database from the US SEER tumor registry database. The SEER tumor registry collected information from about 10% of all breast cancer cases in the USA. The database used for Adjuvant! Online included information such as the patients’ demographics and tumor characteristics (tumor size, the number of positive nodes, tumor grade), and survival in postoperative breast cancer patients aged 20 to 79 years between 1988 and 1992 [11]. After entering these data, the program calculated the annual breast cancer mortality rates and produced data for comparison with the database from the SEER tumor registry. These data were used to predict the 10-year survival. Adjuvant! Online can be used to provide recommendations for adjuvant systemic therapy after considering the estimated 10-year overall survival (OS), breast cancer-specific survival (BCSS), and event-free survival (EFS) However, the lack of prognostic power of Adjuvant! Online in different populations raises questions about the combination of prognostic factors and accuracy of patients’ characteristics. Our goal was to determine the accuracy of the program applied to an Asian population and, if the program is accurate, which subgroup should be included.

The tool has been validated and used by oncologists in different countries including Canada, Germany, Holland, and the United Kingdom (UK) [12,16-19]. In an analysis of 4083 early breast cancer patients in Canada, Olivotto et al. showed that the overall predicted and observed 10-year outcomes were within 2% for OS, BCSS, and EFS [12]. The Adjuvant! Online program was also validated in small cohorts of patients in Germany [19]. The increased use of the program by physicians and the positive results in Western countries prompted us to analyze the accuracy of the program in an Asian population.

In the present study, the difference between the predicted and observed outcomes in the low-risk cohort was about 1%. We conclude that Adjuvant! Online is an accurate tool for predicting the outcome in low-risk breast cancer patients in the Taiwanese population. By contrast, we observed a large discrepancy between our prediction and that of Adjuvant! Online in the high-risk population. That is, Adjuvant! Online underestimated the mortality risk in the high-risk subgroup of Taiwanese breast cancer patients. Considering this discrepancy, we suggest that a correction factor of 1.259 might be justified for high-risk patients. Variations in the program validation may differ between countries and ethnic factors are known to be determining factors that can influence the decision about and outcomes of adjuvant treatment. The program should be validated in different countries and ethnic groups before wider application of these data.

Campbell et al. showed that the recommendations for adjuvant treatment made by a UK-based multidisciplinary team using Adjuvant! Online would improve decision making in only a minority (2.5%) of patients [16]. Our results are similar to those of Campbell et al. In a cohort of 1065 British early breast cancer patients, Campbell et al. found that Adjuvant! Online significantly overestimated OS (5.54%, *p* < 0.001), BCSS (4.53%, *p* < 0.001), and EFS (3.51%, *p* = 0.001). They concluded that overestimation of BCSS implies that Adjuvant! Online underestimates breast cancer mortality for women in the UK. One possible reason for this difference may be that the breast cancer mortality rate is lower in the USA than in the UK.

A Dutch validation study of 5380 breast cancer patients showed no significant differences between the 10-year observed OS and BCSS compared with the Adjuvant! Online-predicted OS and BCSS (*p* = 0.87 and *p* = 0.18, respectively) [20]. However, in the subgroup with patients aged < 40 years, Adjuvant! Online overestimated the predicted OS and BCSS (4.2%, *p* = 0.04 and 4.7%, *p* = 0.01, respectively). In the patient subgroup older than 69 years, Adjuvant! Online overestimated the predicted OS (3.4%, *p* = 0.05). Finally, the program underestimated predicted BCSS in the patient subgroup with 1–3 lymph node metastases (3.1%, *p* = 0.002). Our data are consistent with these earlier findings in that we found that Adjuvant! Online predicted BCSS accurately in some patient subgroup.

Compared with other studies that validated the accuracy and performance of Adjuvant! Online, our study might be considered to have had a small sample size. However, one advantage in our study is the low proportion of missing data. Data for tumor size, lymph node status, ER status, and tumor grade were collected routinely and rechecked by a clinician and data manager, and our missing data rate was around 25%. Our missing data rate is similar to the publication from Bhoo-Pathy et al. (25%) [21]. In the study by Mook et al., 3104 of 5380 patients (58%) had data missing for ER status or tumor grade. However, they concluded that including the patients with incomplete data did not influence the prediction of outcomes. The program can also predict disease outcome without information about ER status. By contrast, Adjuvant! Online tend to underestimate the BCSS in ER-negative breast cancer patients. Mook et al. concluded that this result was observed in patients undergoing hormonal treatment [20]. Another limitation of our study is the adjuvant treatment modalities. The period of data recruitment for this study was from 1992 to 2001. According to the NCCN guidelines and policy of Taiwan national health insurance, standard adjuvant chemotherapy comprised CMF (cyclophosphamide, methotrexate, and fluorouracil) or an anthracycline-based regimen, and standard adjuvant endocrine therapy comprised tamoxifen for HR-positive patients. However, the treatment varies between countries, especially in relation to different national policies for the use of treatments, such as taxane-containing regimen chemotherapy or aromatase inhibitors.

## Conclusions

This validation study of Adjuvant! Online applied to a university hospital-based cohort of Taiwanese breast cancer patients showed that the calculated predictions by Adjuvant! Online matched the observed outcomes and that the predictions are suitable when applied to low-risk, early breast cancer patients. We agree that Adjuvant! Online is a useful tool for doctors and patients, but it should be used only by those aware of its limitations, as advised in the program’s instructions and as reported elsewhere.

## Competing interests

The authors declare that they have no competing interests.

## Authors' contributions

KYL and CTW designed the concept of this study, drafted the manuscript and performed treatment. CTW and KYL collected the data and performed the statistical analysis. KYL, CDR and CTW approved the final manuscript. KYL, CDR and CTW designed the concept of this study and provided treatment coordination. All authors read and approved the final manuscript.

## Pre-publication history

The pre-publication history for this paper can be accessed here:

http://www.biomedcentral.com/1472-6947/12/108/prepub
